# Antimicrobial properties of selected microalgae exopolysaccharide-enriched extracts: influence of antimicrobial assays and targeted microorganisms

**DOI:** 10.3389/fmicb.2025.1536185

**Published:** 2025-01-28

**Authors:** Marion Pointcheval, Anthony Massé, David Floc’hlay, Franck Chanonat, Jacques Estival, Marie-José Durand

**Affiliations:** ^1^Nantes Université, Oniris, CNRS, GEPEA, UMR 6144, La Roche-sur-Yon, France; ^2^Nantes Université, Oniris, CNRS, GEPEA, UMR 6144, Saint-Nazaire, France; ^3^Holcim PRB Company, R&D Department, Les Achards, France

**Keywords:** antimicrobial, diffusion assay, exopolysaccharides, growth inhibition, microalgae, microbroth assay

## Abstract

Exopolysaccharide (EPS)-enriched extracts derived from microalgae exhibit a wide range of bioactive properties, including antibacterial and antifungal properties. However, these properties vary depending on the microalgae species, the antimicrobial assay used, and selected targeted microorganisms. This study offers to investigate the antimicrobial properties of exopolysaccharide-enriched extracts obtained from five microalgae species scarcely studied in this context. The targeted microorganisms selected for this study included Gram-positive (*Bacillus subtilis*) and Gram-negative bacteria (*Pseudomonas aeruginosa*), fungi (*Cladosporium cladosporioides*), and microalgae (*Chlorella vulgaris*). Well-diffusion assay, broth microdilution assay, and growth measurements using absorbance were used to compare the methods and fully estimate the antimicrobial properties. Using absorbance measurements, growth rate inhibitions of at least 80% were observed on all targeted species for at least one microalgal EPS-enriched extract. At a concentration of 500 mgGlcEq · L^−1^, most active extracts of *B. subtilis* were obtained from *Chlamydomonas reinhardtii* (87.1% of growth inhibition), *Nostoc commune* (53.7%), and *Eustigmatos polyphem* (46.4%). EPS-enriched extracts from *C. reinhardtii* (86.2%), *N. commune* (59.9%), and *Porphyridium cruentum* (31.1%) were found to be the most effective against *P. aeruginosa*. Antifungal activities were the highest for EPS extracts from *Microchloropsis gaditana* (86.0%), *C. reinhardtii* (16.6%), and *E. polyphem* (17.8%). The results indicated microalgae growth inhibition by EPS-enriched extracts from *N. commune* (99.3%), *C. reinhardtii* (84.8%), and *M. gaditana* (84.1%). To our knowledge, this study is the first to explore the algicidal properties of EPS-enriched extracts derived from microalgae, identifying promising candidates for future investigations into their potential applications.

## 1 Introduction

Microalgae represent a highly taxonomically diversified group of eukaryotic and prokaryotic photosynthetic microorganisms. While predominantly studied over the past decades for their potential in lipid production, microalgae are also a rich source of various compounds such as proteins, pigments, and polysaccharides. These compounds can either accumulate within the cells or be secreted into their surrounding environment (Babiak and Krzemińska, 2021). The production and release of these extracellular substances are often associated with responses to biotic or abiotic stresses, intercellular communication, or biofilm development (Liu et al., 2016). Although polysaccharides and proteins constitute the primary components of extracellular substances, other compounds such as fatty acids, nucleic acids, humic acids, and amino acids have also been detected in smaller amounts (Babiak and Krzemińska, 2021).

Exopolysaccharides (EPS) secreted by microalgae cells exhibit significant structural and compositional diversity, which depends on the species and growth conditions (Angelis et al., 2012; Gaignard et al., 2019; Laroche, 2022). These microalgal EPSs have demonstrated a range of bioactive properties, including antiviral (Huang et al., 2001; Yim et al., 2004), anti-tumor (Gargouch et al., 2021; Sun et al., 2012), anti-cholesterol (Dvir et al., 2000), anti-biofilm (Gargouch et al., 2021), antifungal (Najdenski et al., 2013), and antibacterial activities (Bashir et al., 2018). The antimicrobial properties of microalgal EPS highlight their potential applications in pharmaceutical, agricultural, or aquaculture industries (Stirk and van Staden, 2022).

Previous studies have demonstrated the antimicrobial properties of microalgal EPS-enriched extracts against bacterial or fungal species, employing various methods such as agar diffusion and broth microdilution assays (Bashir et al., 2018; Gargouch et al., 2021; Gigova et al., 2012; Raposo et al., 2014; Najdenski et al., 2013). While these studies have highlighted the potential of EPS extracts, differences in microalgal species, target microorganisms, and assay types make it challenging to directly compare their findings. In this study, we aimed to provide a more standardized and comprehensive approach by assessing the antimicrobial properties of EPS-enriched extracts from microalgae, particularly for environmental applications. Thus, cultivated microalgae in this study have been carefully selected as well as targeted microorganisms included ubiquitous bacteria, fungi, and microalgae species. Finally, to enhance the robustness and diversity of the obtained results, three distinct assays were used to demonstrate potential antimicrobial properties, allowing to address the limitation of using only one or two methods to determine antimicrobial properties for natural extracts.

## 2 Materials and methods

### 2.1 Microalgae cultivation

#### 2.1.1 Microalgal strains and culture conditions

Microalgae species (Table 1) were obtained from the Culture Collection of Algae and Protozoa (CCAP, Edinburgh, Scotland), the Culture Collection of Algae (SAG, Goettingen, Germany), UTEX Culture Collection of Algae (Austin, TX, United States), or AlgoBank Caen (AC, Caen, France). Two sets of culture conditions were used to accommodate both freshwater and seawater microalgal species. Freshwater microalgae were cultivated at pH 7.4 in sterile modified Bold Basal Medium (BBM), while seawater species were cultivated at pH 8.4 in sterile modified Guillard’s F/2 medium. The compositions of the culture media are provided in Supplementary Table S1. Both media were prepared using purified water (Elix^®^ Essential 5, Milli-Q, Merck, Darmstadt, Germany).

**Table 1:** Microalgae species used under study.

Phylum	Microalgae species	Culture medium
Chlorophyta	*Chlamydomonas reinhardtii* CCAP 11/32B	Modified Bold Basal Medium
Cyanophyta	*Nostoc commune* CCAP 1453/33	Modified Bold Basal Medium
Ochrophyta	*Eustigmatos polyphem* SAG 38.84	Modified Bold Basal Medium
	*Microchloropsis gaditana* CCAP 849/5	Modified Guillard’s F/2 Medium
Rhodophyta	*Porphyridium cruentum* UTEX 161	Modified Guillard’s F/2 Medium

Strains were maintained in Erlenmeyer flasks (500 mL) in an incubator (Innova 42, New Brunswick Scientific, Enfield, CT, United States) at 23°C and 130 rpm under continuous light (30 μmol photons·m^−2^·s^−1^). Cultures were grown in a 3 L air-lift column photobioreactor (NANO, Synoxis Algae, Le Cellier, France) with the respective medium at 25°C (±2°C), under a maximum light intensity of 300 μmol photons·m^−2^·s^−1^ and a 12:12 photoperiod. All microalgae species were harvested after 15 days of cultivation, except for *Eustigmatos polyphem*, which exhibited a longer lag phase and was harvested after 20 days. Microalgae cultivations were replicated once for each species. Cultures were harvested by centrifugation (10,000*g* for 10 min at 20°C). Cell-free supernatants were used for the obtention of EPS-enriched extracts, while cell biomass was freeze-dried at −50°C and 0.011 mbar for 48 h before being weighed. Biomass dry weight concentration was determined as follows (Equation 1):


(1) $$C_{\mathrm{final}\;\mathrm{biomass}}\left(gDW.L^{-1}\right)=\frac{m_{\mathrm{final}\;\mathrm{biomass}}}{V_{\mathrm{final}\;\mathrm{culture}}}$$

where *m_final biomass_* is the final biomass weight obtained after cultivation (gDW), and *V_final culture_* is the final volume of the culture (L).

#### 2.1.2 Growth monitoring

Microalgae growth was monitored using light absorbance at 750 nm with a UV–visible spectrophotometer (SAFAS UVmc2, Monte Carlo, Monaco). Samples were diluted to maintain absorbance values below 0.8. Absorbance measurements were performed in triplicate.

As biomass dry weight concentration (gDW · L^−1^) was used for the construction of growth curves, relationships between absorbance and dry weight were established following the protocol outlined by Ratha et al. (2016). Whatman GF/C (47 mm diameter, 1.2 μm pore size) filter papers were dried overnight at 60°C. Then, filter papers were placed in a vacuum desiccator for 30 min and weighed using an analytical balance (Practum 124, Sartorius, Göttingen, Germany) with a resolution of 0.0001 g. Microalgae were cultivated in Erlenmeyer flasks for 15 to 20 days in appropriate culture media. Cultures were 2-fold serially diluted, absorbance at 750 nm for each solution was measured, and a volume of 10 mL was filtered under vacuum through pre-dried and pre-weighed filters. After filtration, the filter papers were dried for 48 h at 60°C before being weighed, and biomass concentrations in samples were obtained.

Relationships between algal dry weight (gDW · L^−1^) and absorbance at 750 nm were described using individual equations constructed for each species. Microalgae-specific growth rate (μ_microalgae_) during the exponential growth phase was calculated according to Equation 2 (Lavrinovičs et al., 2021):


(2) $$\mu_{\mathrm{microalgae}}\left(d^{-1}\right)=\frac{\ln\left({A_{750\;\mathrm{nm}}}_{1}\right)-\ln\left({A_{750\;\mathrm{nm}}}_{0}\right)}{t_{1}-t_{0}}$$

where ${A_{750\;\mathrm{nm}}}_{0}$ and ${A_{750\;\mathrm{nm}}}_{1}$ are the measured absorbance values at time *t*_0_ (initial time) and *t*_1_ (final time), respectively (in days).

### 2.2 Obtention and characterization of EPS-enriched extracts

#### 2.2.1 Extraction of EPS

Extracts were obtained from cell-free supernatants using ethanolic precipitation, as adapted from Ziadi et al. (2018). A volume of 500 mL of cell-free supernatant was mixed with 96% of absolute ethanol (Supelco, Merck, Darmstadt, Germany) in a 1:1 (v/v) ratio and incubated for 24 h at 4°C. The mixture was then centrifuged at 8,048*g* for 10 min at 4°C, and the pellet was rinsed twice with 10 mL of 70% ethanol. The obtained pellets were freeze-dried overnight at −50°C and re-dissolved in sterile distilled water at a final concentration of 25 g · L^−1^.

#### 2.2.2 Characterization of EPS-enriched extracts

##### 2.2.2.1 Carbohydrate content in the extracts

The EPS concentration in the extracts (C_EPS_) was determined using sulfuric acid, as described before (Albalasmeh et al., 2013). This concentration is exclusively used in the rest of this study when referring to concentrations of EPS-enriched extracts and expressed in mg · L^−1^ of D-glucose equivalent (GlcEq). A volume of 1 mL of 10-fold diluted extract was mixed with 2 mL of concentrated sulfuric acid (96%). The mixture was vortexed for 30 s and placed in ice. Absorbance at 375 nm was measured, and EPS concentration (mg · L^−1^) was estimated using a calibration curve established with various D-glucose concentrations. The experiment was performed in triplicate. Carbohydrate content in the extracts was obtained as follows (Equation 3):


(3) $$\mathrm{Carbohydrate}\;\mathrm{content}\;\left(\%m/m\right)=\frac{m_{EPS}}{m_{\mathrm{extract}}}\times 100$$

where *m_EPS_* is the EPS mass (g) determined in the extract, and *m_extract_* is the mass of the extract (g).

##### 2.2.2.2 Protein concentration in the extracts

Protein concentrations were determined using the Pierce BCA Protein Assay Kit (Thermo Scientific, Waltham, MA, United States), as described elsewhere (Felz et al., 2019). Protein content in the extract was calculated as follows (Equation 4):


(4) $$\mathrm{Protein}\;\mathrm{content}\;\left(\%m/m\right)=\frac{m_{\mathrm{protein}}}{m_{\mathrm{extract}}}\times 100$$

where *m_protein_* is the protein mass (g) determined in the extract, and *m_extract_* is the mass of the extract (g).

##### 2.2.2.3 Salt concentration in the extracts

Salt concentrations in the extracts were estimated using a calibration curve established between conductivity (HQ40D conductivity meter, HACH, Loveland, CO, United States) and various NaCl concentrations. Concentrations were expressed in equivalent g · L^−1^ of NaCl (NaClEq).

### 2.3 Determination of EPS concentration in the culture media

The final EPS concentration (Cm_EPS_) in the culture media at the end of cultivation was calculated using Equation 5 and was expressed in mg GlcEq · L^−1^.


(5) $$Cm_{EPS}\left(\mathrm{mgGlcEq}.L^{-1}\right)=\left(C_{EPS}.V_{EPS}\right).\frac{1,000}{V_{\mathrm{culture}}}$$

where *C_EPS_* is the EPS concentration in the extract (mgGlcEq · L^−1^), *V_Extract_* is the volume of the extract (L), and *V_culture_* is the volume of microalgae cell-free supernatant used for extraction (L).

### 2.4 Determination of EPS productivity during cultivation

The specific exopolysaccharide productivity (P_EPS_) was estimated for comparison between species (Laroche, 2022) using Equation 6.


(6) $$P_{EPS}\left(\mathrm{mgGlcEq}.gDW^{-1}\right)=\frac{Cm_{EPS}}{C_{\mathrm{final}\;\mathrm{biomass}}}$$

where *Cm_EPS_* in the EPS concentration in culture media at the end of cultivation (mgGlcEq · L^−1^), and *C_final biomass_* is the final microalgal biomass concentration obtained at the end of cultivation (gDW · L^−1^).

### 2.5 Measurements of antimicrobial activities

#### 2.5.1 Targeted species

The targeted bacterial strains were *Pseudomonas aeruginosa* CIP A22 and *Bacillus subtilis* CIP 52.65 provided by the Institute Pasteur Collection (Paris, France). Bacteria were stored at −80°C in Nutrient Broth (Merck, Darmstadt, Germany) supplemented with 25% (v/v) sterile glycerol. The targeted fungal strain was *Cladosporium cladosporioides* DSM 62121 provided by the Leibniz Institute (Braunschweig, Germany). The fungus was preserved in Petri Dishes containing Malt Extract Agar (Merck, Darmstadt, Germany), in an incubator at 25°C. The microalgae strain chosen for antimicrobial determination was *Chlorella vulgaris* and was maintained as described in Section 2.1. The species tested in this study were selected because they are ubiquitous microorganisms commonly found in the environment, such as subaerial biofilms.

#### 2.5.2 Estimation of antibacterial and antifungal activities

The antibacterial and antifungal activities were estimated using the agar well-diffusion method as described elsewhere (Bashir et al., 2018). The targeted bacterial strains were cultured under agitation (130 rpm) on Mueller Hinton Broth (Merck, Darmstadt, Germany) overnight at 35°C in 50 mL Erlenmeyer (10 mL culture volume). The cultures were then diluted in the fresh identical medium to obtain a bacterial suspension adjusted to McFarland 0.5 standard (approximately 1 × 10^8^ CFU · mL^−1^). Spores from *C. cladosporioides* were recovered by adding a volume of 2 mL of sterile water supplemented with 0.1% Tween 20 on 7-day-old colonies and carefully rubbing with a sterile loop. The spore-containing suspension was then diluted in sterile distilled water to obtain a concentration of 2 × 10^6^ spores · mL^−1^ using a Malassez counting chamber.

The bacterial or fungal suspension was plated on agar layers into 90 × 15 mm Petri Plates containing Mueller Hinton Agar or Malt Extract Agar, respectively (with approximately 25 mL of agar medium per plate). Four wells (8 mm diameter) were prepared in each plate, and a volume of 100 μL of EPS-enriched extracts at a final EPS concentration (C_EPS_) of 1,000 mgGlcEq · L^−1^ was poured into each well. Plates were incubated at 35°C for 24 h for bacterial strains or 25°C for 5 days for the fungal strain. Inhibition zone diameters (mm) were measured using a caliper. Experiments were repeated twice; distilled water and Mergal 712^®^ (Arxada, Basel, Switzerland) at 1% were used as negative and positive controls, respectively.

#### 2.5.3 Determination on MIC of bacterial and fungal targeted strains

Bacterial suspensions described before were adjusted to approximately 1 × 10^5^ CFU · mL^−1^. The fungal suspension was diluted in RPMI 1640 culture medium (Sigma-Aldrich, Saint-Louis, MO, United States) supplemented with 2% glucose and buffered with MOPS (Sigma-Aldrich, Saint-Louis, MO, United States), to obtain a concentration of 2 × 10^5^ spores · mL^−1^, as recommended by the European Committee on Antimicrobial Susceptibility Testing (EUCAST). Suspensions were added to a transparent 96-well cell culture plate (100 μL) and checked against EPS-enriched extracts (100 μL) at final EPS concentrations (C_EPS_) of 50 and 500 mgGlcEq · L^−1^. Microplates were incubated at 37°C for 24 h for bacterial strains or 25°C for 48 h for the fungal strain. The minimum inhibitory concentration (MIC) was estimated visually after incubation. Experiments were repeated twice for consistency; distilled water and Mergal 712^®^ 0.1% were used as negative and positive controls, respectively.

#### 2.5.4 Measurement of bacteria, fungi, and microalgae growth rate inhibitions

##### 2.5.4.1 Preparation of targeted species suspensions

Bacterial (1 × 10^5^ CFU · mL^−1^) and fungal (2 × 10^5^ spores · mL^−1^) suspensions used for the measurements of growth rate inhibitions were prepared as previously described (Section 2.5.2). The targeted microalgae *C. vulgaris* was cultivated in BBM for 7 days, until they reach the stationary growth phase, as verified by absorbance measurements at 750 nm. The culture was then diluted in BBM to achieve a final targeted algal suspension of approximately 1 × 10^5^ cells · mL^−1^.

##### 2.5.4.2 Microorganism incubation and absorbance measurements

A volume of 100 μL of the tested microorganism suspensions was added to the wells of a white 96-well microplate with a transparent bottom. EPS-enriched extracts were then added (100 μL) to the wells, resulting in final EPS concentrations (CEPS) of 0, 50, and 500 mgGlcEq · L^−1^. Positive controls were Mergal 712^®^ 0.1% (Arxada, Basel, Switzerland) for targeted bacteria and fungi species and Preventol^®^ A 6-M (Leverkusen, Germany) for targeted microalgae species. Different NaCl concentrations in sterile distilled water, corresponding to salt concentrations in EPS-enriched extracts, were used as negative controls. Final salt concentrations ranged between 0.09 gNaClEq · L^−1^ for EPS-enriched extracts obtained from microalgae cultivated in modified BBM and 1.55 gNaClEq · L^−1^ for microalgae cultivated in modified Guillard’s F/2 medium.

Microplates containing bacterial and fungal suspensions were incubated as described previously, while microplates with microalgae species were placed in an F-1200 incubator (HiPOINT, Kaohsiung, Taiwan) at 25°C for 72 h under continuous light (50 μmol photons · m^−2^ · s^−1^).

Bacterial growth was measured using light absorbance at 600 nm after 24 h of incubation, while absorbance at 540 nm was used to measure fungal growth after 48 h of incubation. Microalgae growth was quantified with light absorbance at 750 nm after 72 h incubation. Absorbance was also measured at the initial time (A_0_) in sterile culture media. Experiments were conducted twice, with each condition tested in triplicate.

##### 2.5.4.3 Determination of growth rate inhibitions

Growth rate inhibitions were determined according to Organization for Economic Co-operation and Development (OECD) guideline 201, as follows:

The average specific growth rate (*μ_targeted_*) for each targeted strain after incubation was calculated using Equation 7.


(7) $$\mu_{\mathrm{targeted}}\left(h^{-1}\right)=\frac{\ln\left(A_{1}\right)-\ln\left(A_{0}\right)}{t_{1}-t_{0}}$$

where *A*_0_ and *A*_1_ are absorbance values at time *t*_0_ (initial time) and *t*_1_ (final time), respectively (h).

Moreover, global growth rate inhibition (*I_targeted_*) for each extract was calculated according to Equation 8.


(8) $$I_{\mathrm{targeted}}\;\left(\%\right)=\frac{\mu_{\mathrm{negative}\;\mathrm{control}}-\mu_{\mathrm{extract}}}{\mu_{\mathrm{negative}\;\mathrm{control}}}$$

where *μ_extract_* and *μ_negative control_* are the growth rates of targeted microorganism with the tested extract and negative control, respectively (h^−1^).

##### 2.5.4.4 Statistical analysis

Data were analyzed statistically to determine the degree of significance using one-way analysis of variance (ANOVA) at a *p*-value of ≤0.05. Statistical analysis was carried out using R software version 4.4.3.

## 3 Results

### 3.1 Characterization of the microalgae cultures and EPS-enriched extracts

**Figure 1:**
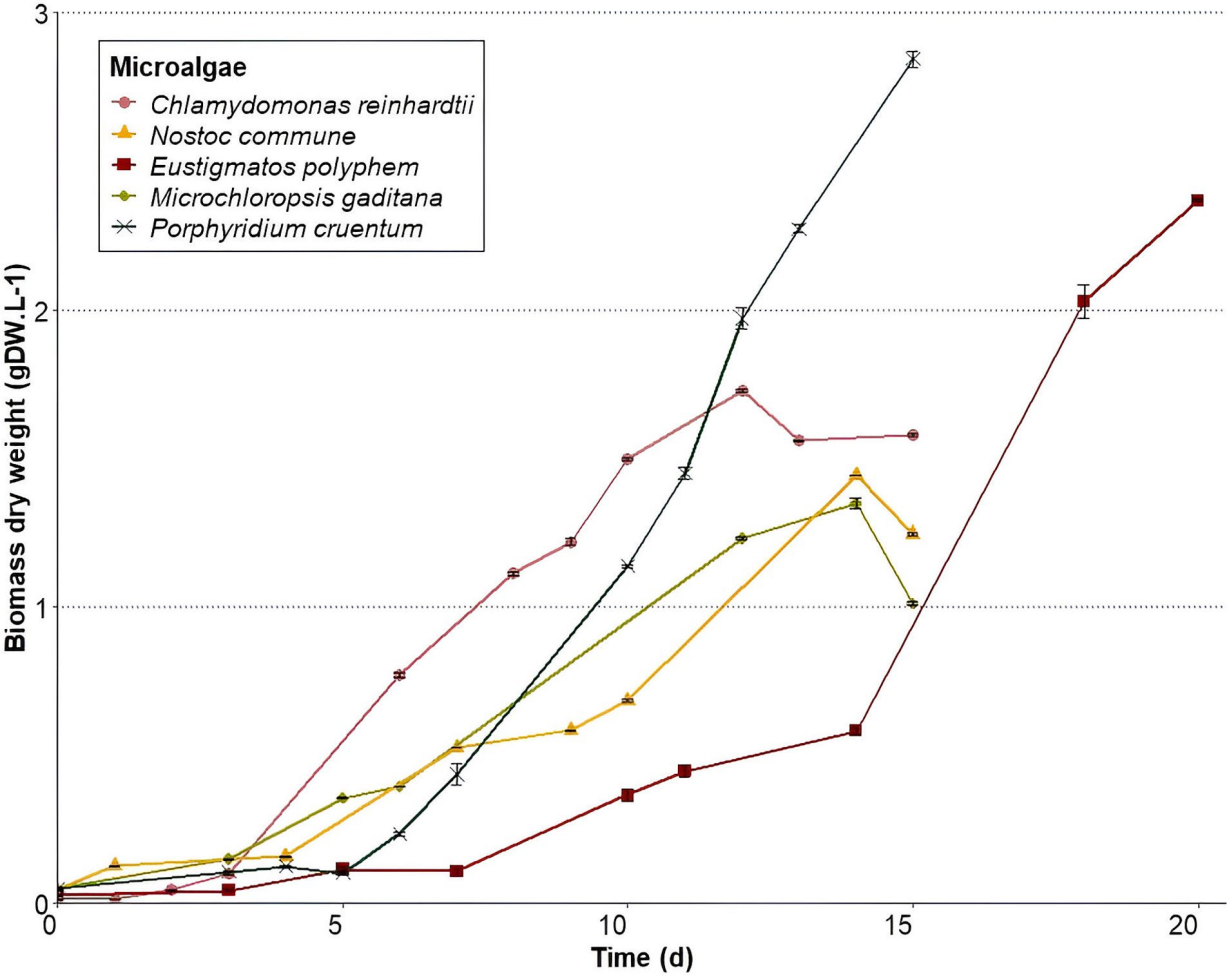
Microalgae growth curves representing biomass dry weight average concentrations ± standard deviation (*n* = 3) over time during cultivation. Microalgae were harvested at the end of respective growth curves.

Microalgae growth rates (Figure 1) were the highest for *P. cruentum* (0.332 d^−1^) and the lowest for *M. gaditana* (0.104 d^−1^) (Table 2). *E. polyphem* and *C. reinhardtii* showed growth rates of 0.302 d^−1^ and 0.228 d^−1^, respectively, while *N. commune* presented a growth rate of 0.193 d^−1^.

**Table 2:** Microalgae-specific growth rates (μ_microalgae_), EPS concentrations in culture media (Cm_EPS_), and EPS productivity (P_EPS_).

Microalgae species	μ_microalgae_ (d^−1^)	Cm_EPS_ (mgGlcEq · L^−1^)	P_EPS_ (mgGlcEq · gDW^−1^)
*Chlamydomonas reinhardtii*	0.228	53.88 (± 1.00)	30.96 (± 0.19)
*Nostoc commune*	0.193	34.89 (± 0.20)	19.60 (± 0.04)
*Eustigmatos polyphem*	0.302	32.48 (± 1.52)	13.27 (± 0.21)
*Microchloropsis gaditana*	0.104	183.28 (± 1.63)	213.12 (± 0.63)
*Porphyridium cruentum*	0.332	855.28 (± 27.26)	268.53 (± 2.85)

Given values are means ± standard deviation (*n* = 3).

EPS final concentrations in culture media (Cm_EPS_) were the highest for *P. cruentum* (855.28 mgGlcEq · L^−1^) and *M. gaditana* (183.28 mgGlcEq · L^−1^). Other species achieved final concentrations of 32.48, 34.89, and 53.88 mgGlcEq · L^−1^ for *E. polyphem*, *N. commune,* and *C. reinhardtii, respectively.*

Specific EPS productivity (P_EPS_) was comprised of between 13.27 and 268.53 mgGlcEq · gDW^−1^ for *E. polyphem* and *P. cruentum,* respectively. Productivities for *N. commune*, *C. reinhardtii,* and *M. gaditana* were 19.60, 30.96, and 213.12 mgGlcEq · gDW^−1^, respectively.

EPS contents in extracts were the highest for *M. gaditana* (48.72%), *E. polyphem* (43.43%), and *P. cruentum* (39.96%) while being lower for *C. reinhardtii* and *N. commune* (4.49 and 1.94%, respectively) (Table 3). No protein was detected for *N. commune* final extract, while 0.03% was found for *C. reinhardtii* and 0.32% for *P. cruentum*. The highest protein contents were found for *E. polyphem* (6.35%) and *M. gaditana* (2.41%).

**Table 3:** Carbohydrate and protein contents (% m/m)in different obtained EPS-enriched extracts.

Microalgae species	Carbohydrate content (%m/m)	Protein content (%m/m)
*Chlamydomonas reinhardtii*	4.49 (± 0.32)	0.03 (± 0.02)
*Nostoc commune*	1.94 (± 0.060)	0.00 (± 0.00)
*Eustigmatos polyphem*	43.43 (± 1.31)	6.35 (± 0.07)
*Microchloropsis gaditana*	48.72 (± 2.14)	2.41 (± 0.12)
*Porphyridium cruentum*	39.96 (± 1.12)	0.32 (± 0.06)

Given values are means ± standard deviation (*n* = 3). Methodologies for the obtention of carbohydrate and protein contents are described in Sections 2.2.2.1 and 2.2.2.2, respectively.

### 3.2 Antimicrobial properties

Estimation of antibacterial properties on both bacterial species (*B. subtilis* and *P. aeruginosa*) and the fungal species (*C. cladosporioides*) using well-diffusion assay revealed an absence of growth inhibition zones (data not shown). Using the same protocol, inhibition zone diameters were 15 mm, 14 mm, and 22 mm for *B. subtilis*, *P. aeruginosa,* and *C. cladosporioides,* respectively, with positive control Mergal 712^®^. No CMI values were determined using broth microdilution assay as the bacterial and fungal species grew in all wells except with positive control (data not shown).

**Figure 2:**
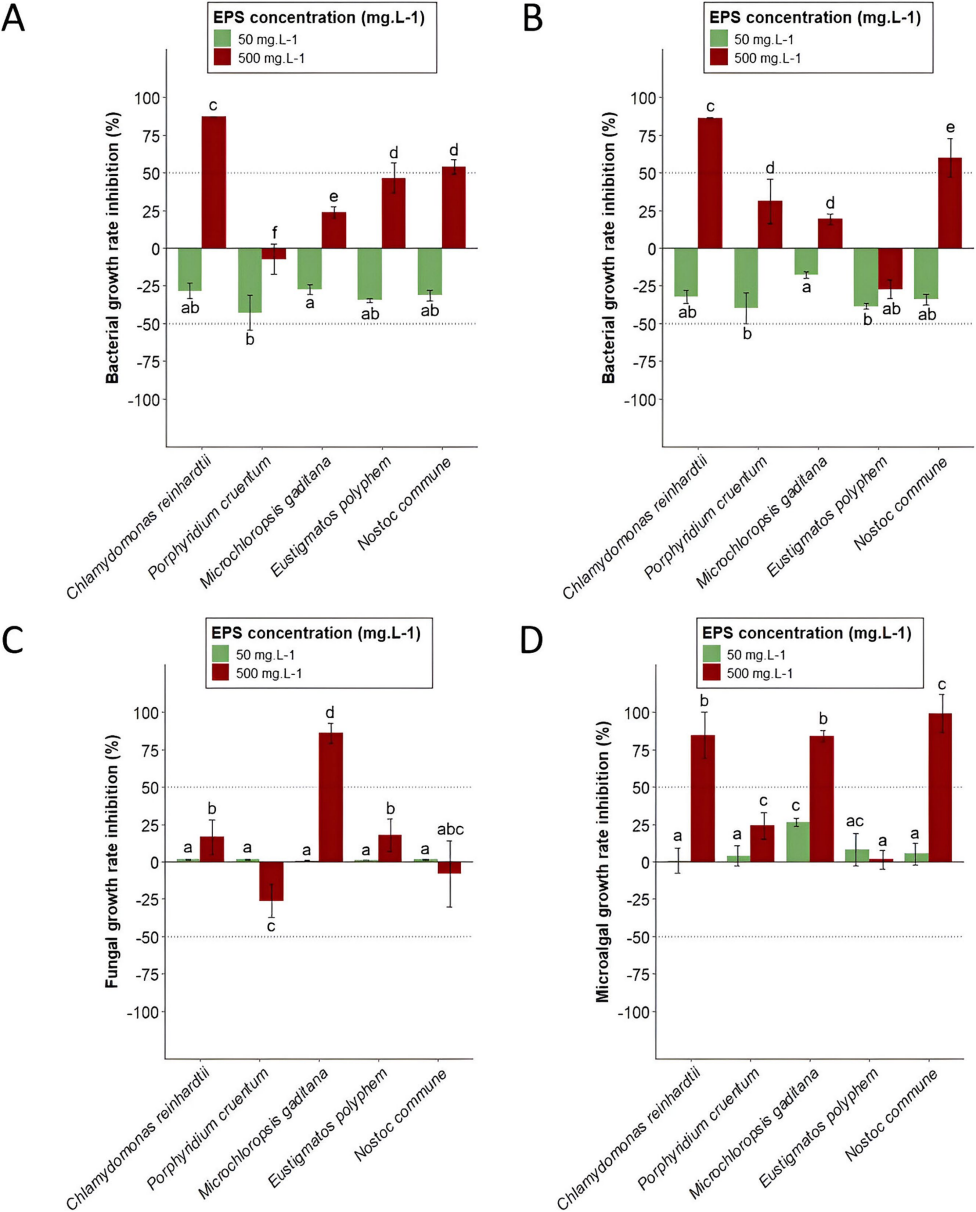
Growth rate inhibitions of microalgae EPS-enriched extracts at different EPS concentrations (50 and 500 mgGlcEq · L^−1^) compared to negative controls (distilled water) obtained using absorbance measurements at the end of incubation for *B. subtilis* **(A)**, *P. aeruginosa* **(B)**, *C. cladosporioides* **(C)**, and *C. vulgaris* **(D)**. Represented values are mean ± standard deviation (*n* = 6). Different letters indicate statistically significant differences using one-way analysis of variance (ANOVA) at a *p*-value of ≤0.05.

As explained in the Materials and Methods part, two concentrations of extracts were tested: 50 mgGlcEq · L^−1^ and 500 mgGlcEq · L^−1^. The first concentration (50 mgGlcEq · L^−1^) led to negative bacterial growth rate inhibitions regardless of the tested extract. Thus, inhibition growth rates of *B. subtilis* were −43.1, −34.7, −31.6, −28.3, and −27.6% for *P. cruentum, E. polyphem, N. commune, C. reinhardtii,* and *M. gaditana* EPS-enriched extracts, respectively (Figure 2A). Negative growth rate inhibition values found against *P. aeruginosa* were −39.7, −38.7, −34.2, −32.5, and −17.8% for *P. cruentum, E. polyphem, N. commune, C. reinhardtii, and M. gaditana,* respectively (Figure 2B). For fungal growth inhibition (*C. cladosporioides*), values of 0.72, 1.02, 1.33, 1.39, and 1.41% were found for *M. gaditana, E. polyphem, P. cruentum, N. commune, and C. reinhardtii* (Figure 2C). Finally, the microalgal growth inhibition (*C. vulgaris*) was 0.76% for *C. reinhardtii*, 3.81% for *P. cruentum,* 5.22% for *N. commune*, 7.98% for *E. polyphem*, and 26.4% for *M. gaditana* (Figure 2D).

**Figure 3:**
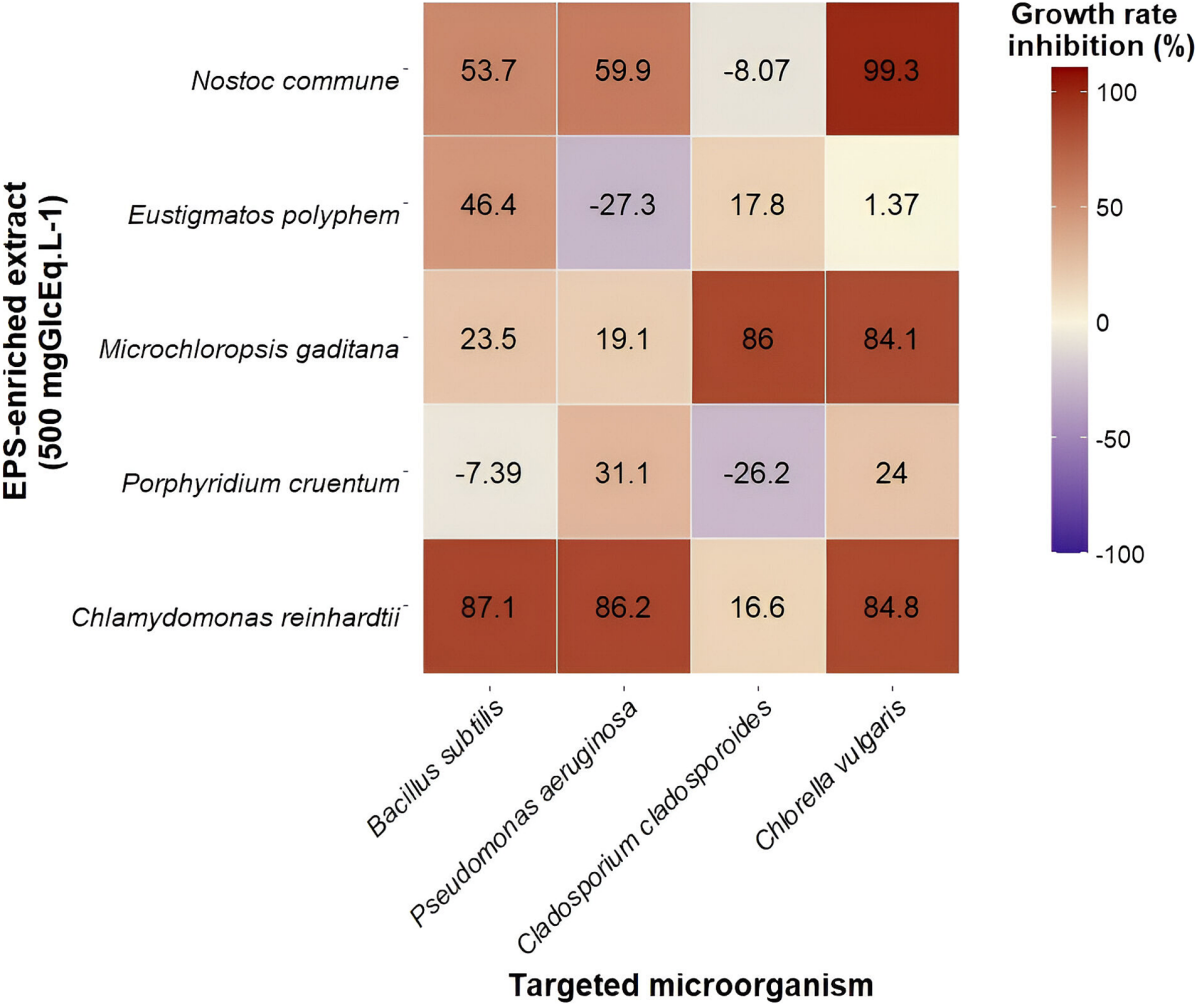
Heatmap summarizing antimicrobial properties of EPS-enriched extracts (at 500 mgGlcEq · L^−1^) obtained from five cultivated microalgae species against four targeted species (bacteria, fungi, and microalgae). Each cell presents the mean growth rate inhibition determined using absorbance measurements (*n* = 6). Dark blue indicates a low growth rate inhibition, while dark red indicates a high growth rate inhibition.

Second, a final EPS concentration of 500 mgGlcEq · L^−1^ was tested. At this EPS concentration, salt concentration ranged between 0.24 and 1.5 gNaClEq · L^−1^ depending on fractions. The obtained growth rate inhibitions, compared to negative controls, are expressed after accounting for the inhibition caused by salts. The highest salt concentration did not impact bacterial growth but reduced *C. cladosporioides* and *C. vulgaris* growth rates by 13.9 and 37.7%, respectively. Thus, bacterial growth inhibitions at this concentration were −7.39, 23.5 46.4, 53.7, and 87.1% for *B. subtilis* (Figure 2A) with EPS-enriched extracts obtained from *P. cruentum*, *M. gaditana*, *E. polyphem*, *N. commune,* and *C. reinhardtii,* respectively. For *P. aeruginosa* (Figure 2B), values were −27.3, 19.1, 31.1, 59.9, and 86.2% with EPS-enriched extracts obtained from *E. polyphem*, *M. gaditana*, *P. cruentum*, *N. commune,* and *C. reinhardtii,* respectively. Fungal growth inhibitions (*C. cladosporioides*) were −26.2, −8.07, 16.6, 17.8, and 86.0% for *P. cruentum, N. commune, C. reinhardtii, E. polyphem,* and *M. gaditana* extracts (Figure 2C). Microalgal growth rate inhibitions (*C. vulgaris*) of 1.37% for *E. polyphem*, 24.0% for *P. cruentum*, 84.1% for *M. gaditana,* 84.8% for *C. reinhardtii,* and 99.3% for *N. commune* were found (Figure 2D). A heatmap was generated to provide an overall view of obtained growth rate inhibitions of all targeted species, for all EPS-enriched extracts (Figure 3) at a final EPS concentration of 500 mgGlcEq · L^−1^.

## 4 Discussion

The obtained results suggest that EPS production by microalgal cells is strongly species-dependent as similar final EPS concentrations in the culture media resulted in different specific growth rates for *N. commune* and *E. polyphem*. However, it is important to consider the difference in harvesting times between the two species, which may influence this observation. Moreover, no correlation was found between algal biomass and EPS concentration. Given that the culture conditions were identical, we concluded that some species are more efficient at producing EPS than others, regardless of growth or cellular concentration. This observation has been demonstrated elsewhere (Gaignard et al., 2019). Commonly, reported values for EPS concentrations in culture media range between 400 and 1,000 mg · L^−1^ (Pierre et al., 2019). Thus, EPS final concentrations obtained in the culture media are lower for all microalgae except *P. cruentum*. However, the production of EPS is highly dependent on culture conditions. For example, *P. cruentum* reached a final EPS concentration in culture media of 1,100 mg · L^−1^ with a MgSO_4_ supplemented culture media compared to 400 mg · L^−1^ with a non-modified media (Raposo et al., 2014). Differences in culture conditions lead to disparity in results, suggesting the need to optimize and standardize culture conditions to achieve maximum EPS concentrations for each species.

EPS content (purity) in the obtained extracts results from the co-precipitation of salt and other impurities, which varies depending on alcohol polarity, temperature, and precipitation duration (Delattre et al., 2016). Thus, EPS content ranged from 12.8 to 33.3% depending on the type of alcohol and extraction time used for *P. cruentum* (Patel et al., 2013). Based on these values, the method used resulted in a high EPS content for *P. cruentum*, *M. gaditana,* and *E. polyphem* but a low EPS content for *N. commune*, *R. subcapitata,* and *C. reinhardtii*. A high salt content was also observed for *M. gaditana* and *P. cruentum*, likely due to the NaCl concentration in the modified Guillard’s F/2 culture medium. Thus, optimization of the recovery method and purification protocols, such as desalting or deproteinization, are necessary to obtain purer extracts (Delattre et al., 2016).

In our study, diffusion assay was ineffective to underlying any antibacterial properties from microalgae. This method relies on the diffusion of compounds through an agar medium to inhibit the growth of targeted microorganisms. While this method was effective for some EPS-enriched extracts (Gargouch et al., 2021), it is highly dependent on the agar medium characteristics, pH, agar depth, and moisture content as these factors impact the diffusion range of tested compounds. In addition, EPS-enriched extracts obtained from microalgae present various rheological behaviors, molecular sizes, and solubilities, which may influence their diffusion abilities in agar (Bubonja-Šonje et al., 2020; Gargouch et al., 2021; Stirk and van Staden, 2022). Therefore, the well-diffusion assay provided false positive results for four out of five tested EPS-enriched extracts that were active using broth microdilution assay (Najdenski et al., 2013). This assay is still widely used as it is a simple and cost-effective method that does not require specialized laboratory equipment. Nevertheless, negative results are incomplete and a more reliable broth dilution assay is recommended for a more precise estimation of antimicrobial activity (Stirk and van Staden, 2022).

Using a microbroth dilution assay, MIC (total visible inhibition) was not reached in our study. With this method, tested compounds are directly in contact with the targeted microorganisms. Thus, this method is more reliable, sensitive, and reproducible than the well-diffusion assay, especially for EPS-enriched extracts, while being also time-consuming and more expensive (Bubonja-Šonje et al., 2020). Therefore, EPS-enriched extracts obtained from *Porphyridium marinum* showed MIC values from 62.5 to 1,000 mg · L^−1^ against *Staphylococcus aureus* and *Escherichia coli,* respectively (Gargouch et al., 2021). Other studies revealed MIC ranging from 140 to 1,000 mg · L^−1^ for various EPS-enriched extracts against different bacterial species (Najdenski et al., 2013). The concentrations used in the literature are generally higher than those in our study (up to 1,000 mg · L^−1^), which may explain why the MIC was not reached in our experiments. Further study focusing on various EPS concentrations is needed to complete the understanding of the antimicrobial activities of the extracts using microbroth dilution assay. Moreover, while the microbroth dilution assay is widely used and standardized, it provides only partial information on growth inhibition as it determines solely total visual growth inhibition. Consequently, the MIC is particularly informative when achieved but remains ambiguous when it is not.

Absorbance is also used for the measurements of growth inhibition from natural compounds (Bouarab-Chibane et al., 2019). The advantage of this method is the fast indication of growth while being simple and inexpensive (Schumacher et al., 2018). In our study, absorbance measurement allowed to unveil the antimicrobial activities of EPS-enriched extracts with a more precise detection limit. A similar absorbance method also determined the antibacterial activity of carrageenan against *Salmonella enteritidis* (Yamashita et al., 2001). This further indicates that both commonly used well-diffusion assay and microbroth dilution assay are limited for the determination of growth inhibition from natural extracts. In addition to antimicrobial properties, absorbance measurements revealed negative growth rate inhibitions of targeted microorganisms in the presence of low EPS concentrations. This phenomenon can be attributed to the degradation and consumption of EPS by the tested microorganisms, as previously described (Zhang et al., 2015).

Thus, this method revealed antimicrobial properties against bacteria, fungi, and microalgae of EPS-enriched extracts that would have remained undiscovered using conventional methods. Absorbance measurements showed significant results concerning antimicrobial properties with variation among targeted species. Such variations can be explained by the different cell wall or cell membrane compositions between Gram-positive and Gram-negative bacteria, fungi, and microalgae. In fact, EPS antibacterial mechanisms are related to the structural disruption of the cell wall and cell membrane or their ability to penetrate the cell (Salimi and Farrokh, 2023). Therefore, no EPS extract was highly effective on all microorganisms in our study as *C. vulgaris* was particularly sensible to EPS-enriched extracts from *C. reinhardtii*, *N. commune,* and *M. gaditana* while *C. cladosporioides* was mostly sensible to *M. gaditana* EPS-enriched extract only. For bacteria species, the most effective EPS-enriched extracts were obtained from *C. reinhardtii*. This last result is in accordance with the study of Vishwakarma and Vavilala (2019) which demonstrated the antibacterial properties of sulfated polysaccharides from *C. reinhardtii*. Thus, this species is especially interesting for the antimicrobial properties of its intracellular and extracellular polysaccharides and requires further study. As our study is the first to focus on algicidal properties from microalgae EPS extracts, no comparison with previous studies is possible. Nevertheless, EPS obtained from the fungi *Fusarium fujikuroi* presented herbicidal activity (Todero et al., 2020); thus, such compounds have potential applications as herbicidal or algicidal agents. Variation between antimicrobial activity is also correlated to the EPS molecular composition in the EPS-enriched extracts and thus to microalgae species and culture conditions. In fact, bioactivity properties have been previously linked to sulfate content (Morais et al., 2022), and such content varies according to species (Delattre et al., 2016) and culture conditions (Raposo et al., 2014).

In conclusion, our study highlighted the limitations of relying on a single method for the determination of antimicrobial properties. The well-diffusion assay exhibited a lack of sensitivity, while the microbroth dilution assay provided only partial information concerning growth rate inhibition. Nevertheless, absorbance measurements enabled the identification of new EPS-enriched extracts (*C. reinhardtii*, *N. commune*, *M. gaditana,* and *E. polyphem*) with antimicrobial against bacteria, fungi, and microalgae species. Therefore, the exclusive uses of these two assays may give false-negative results concerning antimicrobial properties of natural extracts. The use of methods with complementary sensitivities is recommended for upcoming screening studies. Further study regarding optimization of culture conditions, recovery of EPS from culture media, purification, and complete characterization of the extracts is needed as perspectives to increase and fully comprehend the antimicrobial properties of these compounds.

## Data Availability

The raw data supporting the conclusions of this article will be made available by the authors, without undue reservation.
