# Supplementary material for: Antimicrobial properties of selected microalgae exopolysaccharide-enriched extracts: influence of antimicrobial assays and targeted microorganisms
Source: Front Microbiol. 2025 Jan 28;16:1536185. doi: 10.3389/fmicb.2025.1536185 (PMC11813221; doi:10.3389/fmicb.2025.1536185)
Supplement: Supplementary file 1 [file Data_Sheet_1.pdf]

Supplementary Material

Table 1. Compounds concentrations (mM) in both microalgae culture media.

Compounds Name	Modified Bold Basal Medium	Modified Guillard's F/2 Medium
Sodium nitrate	17.6	16.8
Sodium chloride	X	2.1
Magnesium sulfate heptahydrate	9.1×10^{-1}	9.1×10^{-1}
Calcium chloride dihydrate	1.7×10^{-1}	1.7×10^{-1}
Zinc sulfate heptahydrate	7.7×10^{-4}	7.7×10^{-4}
Cobalt nitrate hexahydrate	1.5×10^{-4}	X
Copper sulfate	5.0×10^{-4}	4.0×10^{-4}
Orthoboric acid	4.6×10^{-2}	X
Manganese chloride tetrahydrate	9.2×10^{-3}	9.0×10^{-3}
Sodium molybdate	1.1×10^{-3}	2.5×10^{-4}
EDTA disodium	1.3×10^{-1}	X
Iron sulfate heptahydrate	5.0×10^{-2}	X
Ferric chloride hexahydrate	X	1.2×10^{-1}
Dipotassium phosphate	8.6×10^{-1}	8.6×10^{-1}
Potassium phosphate	9.0×10^{-1}	9.0×10^{-1}
Sodium bicarbonate	1.5×10^{-2}	1.6×10^{-2}
EDTA disodium	1.3×10^{-1}	X
Sodium phosphate dihydrate	X	4.2×10^{-1}
Cobalt (II) chloride hexahydrate	X	4.2×10^{-4}
